# Supplementary figures and images for: Effect of gluten-free diet and antibiotics on murine gut microbiota and immune response to tetanus vaccination
Source: PLoS One. 2022 Apr 13;17(4):e0266719. doi: 10.1371/journal.pone.0266719 (PMC9007335; doi:10.1371/journal.pone.0266719)

A

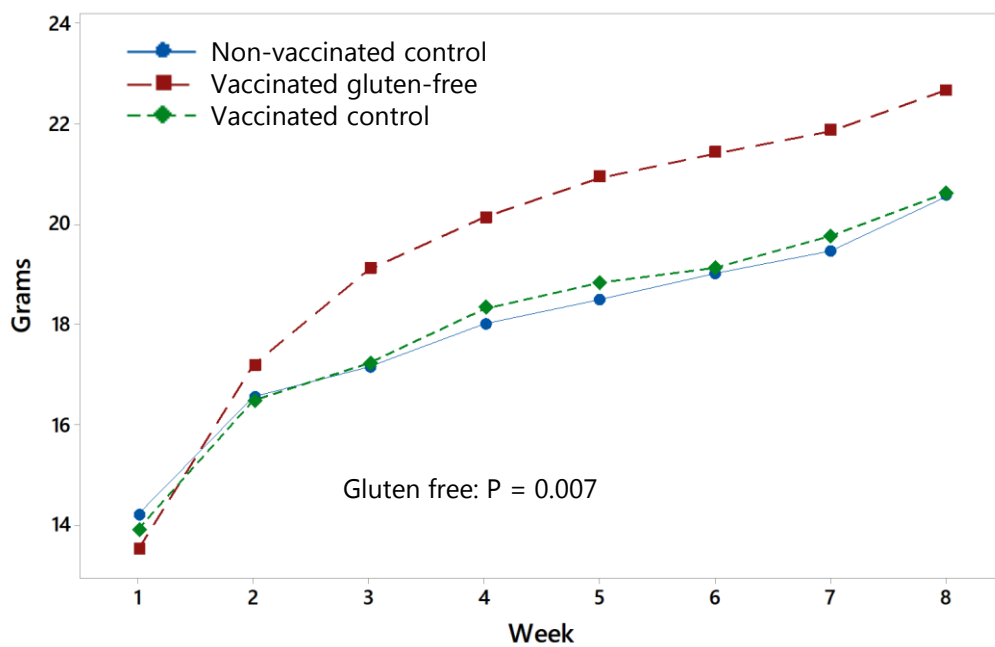

B

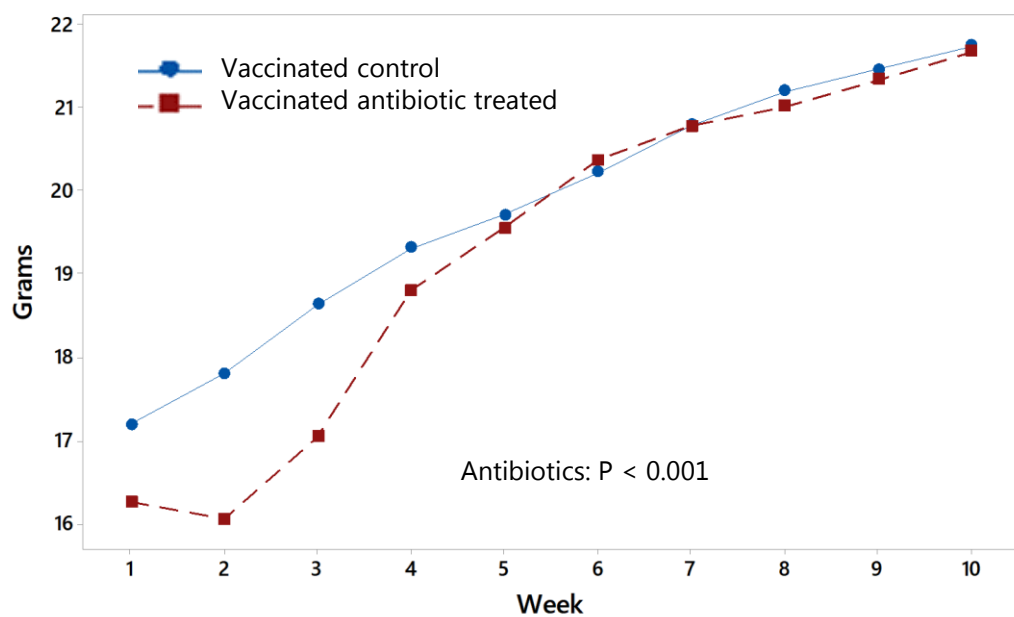

Supplement: S1 Fig — Growth curves for mice fed a gluten free diet (a) or antibiotics (b). (a) BALB/cBomTac mice were vaccinated with a tetanus vaccine (Vaccinated control) or not (Control) and fed a standard Altromin wheat based diet (‘gluten’), or they were vaccinated and fed a modified Altromin diet, in which wheat protein was replaced with casein (‘gluten free’). (b) BALB/cBomTac mice were vaccinated twice with a tetanus vaccine, either in combination with ampicillin (antibiotics) in the drinking water or with pure drinking water (control). Borderline p-values are written in italics. (PDF) [file pone.0266719.s001.pdf]
