# Supplementary material for: Effect of gluten-free diet and antibiotics on murine gut microbiota and immune response to tetanus vaccination
Source: PLoS One. 2022 Apr 13;17(4):e0266719. doi: 10.1371/journal.pone.0266719 (PMC9007335; doi:10.1371/journal.pone.0266719)
Supplement: S1 Table — (PDF) [file pone.0266719.s002.pdf]

S1 Table
Genes for which expressions were monitored by qPCR in the spleens of tetanus vaccinated antibiotics treated and control mice.

Gene code	Accession number	Encoded protein
Rpl13a		60S ribosomal protein L13a
Actb		Beta-actin
Gusb		Beta-glucuronidase
C3		Complement component 3
Cxcl10		C-X-C motif chemokine ligand 10/Interferon gamma-induced protein 10
Foxp3		Forkhead box P3/scurfin
Gusb		Beta-glucuronidase
Hp		Haptoglobin
Ifng		Interferon gamma
Il10rb		Interleukin 10
Il12a		Interleukin 12 subunit alpha
Il18		Interleukin 18
Il1a		Interleukin 1 alpha
Il1beta		Interleukin 1 beta
Il2		Interleukin 2
Klf2		Krüppel-like Factor 2
Klf4		Kruppel-like factor 4
Lrrn3		Leucine-rich repeat neuronal protein 3
Myc		Myelocytomatosis oncogene
Myd88		Myeloid differentiation primary response 88
Nfkb		Nuclear factor kappa-light-chain-enhancer of activated B cells
Nfkbia		NF-κB inhibitor alpha
Nkap		NF-κB activating protein
Tgfb1		Transforming growth factor beta 1
Timp		Tissue inhibitor of metalloproteinases
Tlr1		Toll-like receptor 1
Tlr4		Toll-like receptor 4
Nlrp3		Nucleotide-binding domain leucine-rich repeat containing protein 3/cryopyrin.
Stat4		Signal transducer and activator of transcription 4
