# Supplementary material for: Effect of gluten-free diet and antibiotics on murine gut microbiota and immune response to tetanus vaccination
Source: PLoS One. 2022 Apr 13;17(4):e0266719. doi: 10.1371/journal.pone.0266719 (PMC9007335; doi:10.1371/journal.pone.0266719)
Supplement: S2 Table — No significances were found if P-values were corrected by false discovery rate (Q). (PDF) [file pone.0266719.s003.pdf]

S2 Table

Spleen gene expressions for which a significant difference between tetanus vaccinated antibiotics treated and control mice was found by Kruskall Wallis test (P). No significancies were found if P-values were corrected by false discovery rate (Q).

**Gene code**	**Primer sequence 5' to 3' (F = Forward; R = Reverse)**	**Fold change**		**Kruskall-Wallis test**	**False discovery rate**
		Antibiotics	*Vaccinated controls*	*P*	*Q*
Cxcl10	(F) AGTGCTGCCGTCATTTTCT<br>(R) CCTATGGCCCTCATTCTCAC	1.5445±0.3478	1.8595±0.3208	0.002	0.081
Hp	(F) TATCGCTGCCGACAGTTCTAC<br>(R) CTCTCCAGCGACTGTGTTCA	7.266±4.166	4.573±2.053	0.016	0.215
Ifng	(F) TTTGAGGTCAACAACCCACAG<br>(R) GCTTCCTGAGGCTGGATTC	2.2041±0.4374	1.9033±0.4317	0.048	0.313
Klf2	(F) CGTACACACACAGGTGAGAAGC<br>(F) CTGTGTGCTTTCGGTAGTGG	1.2209±0.1383	1.3618±0.1604	0.008	0.162
Tlr4	(F) CTTCAACCAAGAACATAGATCTGAGC<br>(R) GTCTCCACAGCCACCAGATT	4.149±2.458	4.931±1.778	0.048	0.313
