# Supplementary material for: Effect of gluten-free diet and antibiotics on murine gut microbiota and immune response to tetanus vaccination
Source: PLoS One. 2022 Apr 13;17(4):e0266719. doi: 10.1371/journal.pone.0266719 (PMC9007335; doi:10.1371/journal.pone.0266719)
Supplement: S3 Table — Only correlations significant after FDR correction are shown. (DOCX) [file pone.0266719.s004.docx]

| **S3 Table.** Correlations of taxa with anti-tetanus IgG in BALB/cBomTac mice (n = 40) vaccinated once or twice with a tetanus toxoid vaccine. Only correlations significant after FDR correction are shown. All P and Q values are 0.000. | |
| --- | --- |
| **Taxa** | **Correlation coefficient** |
| **Bacteroidetes** |  |
| Bacteroidia; Bacteroidales; Bacteroidaceae; *Bacteroides*; other | 0.53 |
| Bacteroidia; Bacteroidales; Bacteroidaceae; *Bacteroides*; other | 0.44 |
| Bacteroidia; Bacteroidales; Bacteroidaceae; *Bacteroides*; other | 0.48 |
| Bacteroidia; Bacteroidales; Bacteroidaceae; *Bacteroides uniformis* | 0.53 |
| Bacteroidia; Bacteroidales; S24-7; other; other | 0.48 |
| Bacteroidia; Bacteroidales; S24-7; other; other | 0.47 |
| Bacteroidia; Bacteroidales; S24-7;; other ; other | 0.46 |
| Bacteroidia; Bacteroidales; S24-7;; other ; other | 0.48 |
| **Firmicutes** |  |
| Clostridia; Clostridiales; other; other | 0.47 |
| **Verrucomicrobia** |  |
| Verrucomicrobiae; Verrucomicrobiales; Verrucomicrobiaceae; *Akkermansia muciniphila* | 0.49 |
