# Supplementary material for: Effect of gluten-free diet and antibiotics on murine gut microbiota and immune response to tetanus vaccination
Source: PLoS One. 2022 Apr 13;17(4):e0266719. doi: 10.1371/journal.pone.0266719 (PMC9007335; doi:10.1371/journal.pone.0266719)
Supplement: Attachment — Submitted filename: Rebuttal letter.pdf [file pone.0266719.s005.pdf]

UNIVERSITY OF COPENHAGEN
DEPARTMENT OF VETERINARY AND ANIMAL SCIENCES

Plos One
Editor Dr. Brenda Wilson

**Rebuttal letter**

24 JANUARY 2022

**SECTION FOR EXPERIMENTAL ANIMAL MODELS**

9 RIDEBANEVEJ
2ND FLOOR
DK-1871 FREDERIKSBERG C
DENMARK

TEL 45 35 33 27 26
DIR 45
MOB 45 30 66 34 86

akh@sund.ku.dk

REF: AKH

Dear Brenda Wilson

Thank you very much for once again considering our paper for publication and for inviting yet tow more qualified reviewers.

We feel that these constructive input from the reviewers have improved our manuscript substantially.

Our point-to-point replies are as follows:

**Reviewer #2:**

**Fig5 is problematic, it is not known which two groups are different from each other simply by showing a p-value. The same thing is true for Fig4a,b, and e.**

We understand the concerns of the reviewer, because it is very common to analyse such data by a one-way ANOVA followed by, what in this case would be a Tukey’s post hoc test. However, if the study as in this case is planned accordingly the most powerful way to analyse these data are by a two-way ANOVA, and in a two-way ANOVA you get a p-value for each factor across the groups. We do not find that it is statistically appropriate (although offered by some statistical software packages) to supply it with a post-hoc test, as the beauty of a two-way ANOVA is that you get one p-value for each factor, and attempts to calculate more p-values increases the risk of type I errors without actually adding any value to the evaluation. In addition, the p-values to these factors fully respond to the hypothesis. However, if the editor prefers that we do it, we will of course do it and put it into the table.

**Ampicillin treatment in study B is a little bit too long. Does short-term one-week treatment also have an impact?**

We are a bit uncertain with how the reviewer defines 'too long', but we understand it as if it should be compared to a human situation. In this study ampicillin treatment was used to block microbiota impact on the vaccine response to show if there was a microbiota related effect. It is common practice in antibiotic depletion studies to start out by a depletion for the entire study period, i.e. in this case during the vaccination and until the last antibody monitoring. So, this is what we have done. A short term depletion changes the microbiota afterwards, and we are in the process of studying whether such a change can have long term effects. We do not at present have the basis for further studies on such short term treated animals, and more basic studies of potential immune changes are needed to make out, when to expect changes in a vaccine response. However, we admit that our studies cannot be used to state anything about how the general use of ampicillin/amoxicillin for children affects their immune response; especially not if they are not treated with it at vaccination time point. This should have been better discussed, and we have now introduced the following in the discussion:

*L430-438: In this study, mice were treated with ampicillin throughout the study, which is much longer than humans normally would be treated. During ampicillin treatment the majority of naturally gut inhabiting bacteria will either be eradicated or heavily suppressed, and when ampicillin treatment in mice is terminated, some microorganisms will recover from the host, while other will recover from the environment, and, therefore, ampicillin treated mice will have a microbiota different from non-treated mice [31]. It is unknown whether such a microbiota change will have long term impact on the immune system, and, therefore, our studies cannot be used to state whether the general use of antibiotics in children hampers their responses to vaccines; especially not if they are not treated during vaccination.*

We have also explained this better at the end of the introduction:

*LN99-100: To block the impact of the GM, some of the mice were treated or not treated with ampicillin.*

**Can the author demonstrate that the gluten-free diet-induced microbiota changes drive the lower primary antibody response by fecal transfer type of experiment?**

We agree that this I a perspective for further studies, which has already been mentioned in the discussion ('To verify this further studies should attempt to transfer the GM effect to germ-free mice through a fecal matter

transplantation’). We have a manuscript accepted for Journal of Autoimmunity, in which we in a two-generation show that a gluten-free diet effect is transferred over generations, and some of the effect is microbiota dependent and some is microbiota independent. We have inserted a reference to this:

*L383-393: In humans, it has been shown that gluten-free diet induced GM changes are essential for the increased $T_{reg}$ levels [51], although also a direct anti-inflammatory effect of a gluten-free diet has previously been shown in mice [52]. From studies in two-generation studies in non-obese diabetic mice, it is known that the effects of a gluten-free diet on the immune system can be transferred over generations both dependently and indepently of the GM [15, 53]. In the short term study C it was difficult to verify any major anti-tetanus IgG titer effect of the gluten-free diet one week after vaccination, and the impact of the gluten-free feeding was also minor compared to the two other studies. However, there was still in terms of cell counts a clear impact on the immune system, which therefore either responds to even small GM changes and/or directly to the changed diet composition. Whether the impact on vaccine response is GM dependent might be further elucidated through a fecal matter transplantation to germ-free mice.*

**Can the author demonstrate which cell population influenced by the diet reduced the antibody response?**

FACS data were generated on study C mice, which was too short to show the immediate effects of the first vaccination, as this was when a difference was observed. However, this should have been mentioned in the discussion. We have now written:

*LN387-389: In the short term study C it was difficult to verify any major anti-tetanus IgG titer effect of the gluten-free diet one week after vaccination, and the impact of the gluten-free feeding was also minor compared to the two other studies.*

**The language can be modified further. p10 L223, please delete the ' mark.**

We have made sure that there is no such mark in L223. We have gone through the manuscript with the department translator, and it has be substantially changes. If this from an English language point of view is not enough, we will after acceptance pass it through an editorial service if requested by the editor.

## <u>Reviewer #3:</u>



*The article aims to characterize the impact of microbiome alteration (either through diet or antibiotics) on tetanus vaccine immunogenicity in mice. The article is a nice addition to the literature that suggests that alteration of the intestinal microbiome alters host immune response to both vaccines and pathogens. The authors have performed experiments with a large sample sizes and evaluated microbiome, immune cells, and gene expression resulting in an interesting data set that suggests gluten free-diet and antibiotic administration diminish anti-tetanus antibodies following intial vaccination, possibly through different mechanisms.*

*<u>Major comments</u>*

**The reviewer requests a better rationale for why tetanus vaccine was used for this study, given that it is known to elicit consistently high antibody responses across geography and across ages.**

This is a quite obvious and understandable request from the reviewer. When we planned the study, , we found – as we tried to relate the effect to bacteria of the gut – that it would be most relevant in the first place to study this in a vaccine based upon a bacterial product known to induce a strong Th1 reaction, and therefore we chose the tetanus vaccine. Obviously, in the light of COVID-19 pandemic, it would probably have been relevant to base our studies on a virus vaccine. Having said that we can also see from some of the work we do today that the responses we see in relation to viral vaccines is stronger in their CD8 than in their CD4 reaction, and the knowledge we have built from our work with gluten-free diets over the last decade points in the direction of a gluten-free impact on CD4 rather than CD8.  We have changed the last part of the introduction into:

*LN90-100: We hypothesized that gluten-free diet induced GM perturbations would influence vaccine response in mice. We found it most relevant to study this in relation to a vaccine based upon a bacterial product, and, therefore, we chose to study the impact on a tetanus toxoid vaccine, although this vaccine generally have high immunogenicity and protection globally [39]. Tetanus toxoid is a bacterial product, which when used as vaccine, is known to raise a strong Th reaction [40], and it has traditionally been used with aluminium oxide hydrate adjuvant, which is expected to drive a Th2 rather than a Th1 response [41]. An increase in $T_{reg}$ relative abundance and subsequent IL-10 secretion, as induced by a gluten-free diet, will reduce both Th1 and Th2 and, thereby antibody producing B cell relative abundance [24, 42]. Therefore, we used the production of specific IgG  as primary readout in mice fed a gluten-free or a standard gluten containing diet. To block the impact of the GM, some of the mice were treated or not treated with ampicillin.*

We have also touched upon it in the limitations:

*LN463-471: Finally, these studies were performed with a tetanus toxoid vaccine, which globally in humans is known as a highly efficient vaccine [39], and to which the immune system responds in ways, which can be different from responses to other vaccines. The effectiveness of viral vaccines seems to vary far more. Titers to influenza varies significantly between vaccinated humans in different geographical regions [1], and even the most efficient COVID-19 vaccines do not offer full protection against infection in humans [74]. Therefore, our studies in mice should not be used to question the efficiency of tetanus vaccination in humans, and from a more applied point of view it would be equally or more relevant to study the impact of the GM on such vaccines; especially in the light of the COVID-19 pandemic.*

**Also suggest to reference more of the recent (human and murine) literature on how microbiota alters vaccine immunogenicity.**

Having gone through the references we admit that some of these were somewhat old, and that certain subjects could be better elucidated by some more recent references, which we have now included in the manuscript.

**Study design - Studies A, B and have differing intervals of treatment (antibiotic/diet) and differing timing of prime and boost of vaccination as well as measurement of antibody responses making it difficult to extrapolate results across the studies. The rationale for choices are not clearly described.**

There are some differences, but the way we have presented the study setups make it look more different than it is. In study A and study B mice were vaccinated at 8 weeks of age, but in the previous manuscript we wrote, when they were vaccinated in relation to the arrival. This is, of course, relevant in relation to how long they were fed gluten-free diet or antibiotics, but this was mostly decided by the vendors delivery plans and not by biological considerations. Normally when we do long term feeding/drinking water intake experiments we put the mice on the diet/water at arrival to avoid stressful changes more than once. After study A we discussed whether 2,5 weeks between the two vaccinations was too short and we decided to use four weeks instead. In study C, when we did the vaccinations only once, we wanted to be sure to have mice with a full functioning immune system and, therefore, we decided to give them an extra week before vaccination.

We have introduced the following under materials and methods:

*LN 129-137: We set up three studies in BALB/cBomTac mice, to investigate the influence of gut microbiota manipulations on the response to a tetanus*

*vaccine. Two studies (A and B) to study the impact of either a gluten-free diet or antibiotics on the antibody production after a boosted vaccination regime, and one study (C) to study the immediate impact of one vaccination on immune cells counts in gluten-free fed or antibiotics treated mice. In study A we boosted with a second vaccination after two and a half weeks, while we in study B reconsidered this and did the boosting after four weeks. In study C we used only one vaccination, because the aim was to see the cell counts in relation to our observations after the first vaccination in study A and B, and, therefore, we also sampled the animals only one week later.*

**Analysis – Given the use of antibody titers, request that the authors repeat antibody statistical analyses using transformed data/geometric mean titers.**

We have ranked all titer values and re-analyzed them. Outcomes were not dramatically changed. Graphs have been changed to show medians instead of means.

**Article can often be confusing to read and suggest input from a native English speaker. Some hypotheses in introduction and conclusion suffer from large jumps across subjects without clear delineation of whether data comes from human or animal subjects. Suggest to review and adjust claims accordingly.**

*Reading the manuscript again we fully agree. We have been thoroughly through the paper together with the department translator, and the introduction and the discussion have both been essentially reorganized. If this from an English language point of view is not enough, we will after acceptance pass it through an editorial service if requested by the editor.*

<u>Minor comments</u>

*Abstract*

***Line 26 Wording is confusing, isn't the primary purpose of the study to evaluate the effect of diet ( gluten-free or gluten-containing) and antibiotics (ampicillin or not) on tetanus vaccine immunogenicity? Suggest to adjust wording***

Point taken. It now reads:

*LN26-27: The purpose of this study was to compare the effect of a tetanus vaccine on immunoglobulin G titers and immune cell levels in female BALB/c mice fed either a gluten-free or a gluten containing diet and treated or not treated with ampicillin.*

**Line 28 suggest change 'lowered' to 'was associated with'**

Done

**Line 29 low-responder not defined in abstract or text, suggest to define if this is a statistical outcome or otherwise remove**

All references to 'Low responders' have been removed.

**Line 29 relative abundance or abundance?**

It no reads 'the relative abundance'

**Line 30 remove word massively, change to significantly if this is the case. If hypothesis is that diet altered microbiota which then altered vaccine response, suggest to start with change in microbiota in this sentence**

'Massively' has been replaced with 'significantly'

**Line 30, suggest to remove 'similarly'- suggests that antibiotics altered microbiota similarly to diet?**

'Similarly' has been replaced with 'also'.

**Line 31, add significance for all findings**

'Significantly' has been inserted

**Line 31, define 'boosting'**

'Boosting' has been replaced with 'a second vaccination'

**Line 32, what was definition of" influenced''?**

'Influenced' has been replaced with 'reduced'

**Line 36, suggest to alter sentence, "these results suggest ….."**

Done

*Introduction*

**Line 44 The authors describe diminished vaccine efficacy in low-middle income settings, however tetanus vaccines have never been shown to have diminished efficacy in low or middle income settings. Rather, they have high immunogenicity and good protection globally, with possible higher immunogenicity in low-income settings. Please correct**

This is a general statement, which is indeed true for some vaccines. We have later in the introduction explained our rationale for choosing the tetanus vaccine, and we have inserted a sentence describing that it is generally efficient:

*LN91-98: We found it most relevant to study this in relation to a vaccine based upon a bacterial product, and, therefore, we chose to study the impact on a tetanus toxoid vaccine, although this vaccine generally have high*

*immunogenicity and protection globally [39]. Tetanus toxoid is a bacterial product, which when used as vaccine, is known to raise a strong Th reaction [40], and it has traditionally been used with aluminium oxide hydrate adjuvant, which is expected to drive a Th2 rather than a Th1 response [41]. An increase in $T_{reg}$ relative abundance and subsequent IL-10 secretion, as induced by a gluten-free diet, will reduce both Th1 and Th2 and, thereby antibody producing B cell relative abundance [24, 42].*

**Line 46 Suggest to use the term low-middle income and high income and not 'developing"and developed"as terminology**

Good point. Done.

**Line 48 – all citations have to do with changing vaccine immunogenicity by genetics and age and do not support differential vaccine performance by geography. Please correct**

The references have been replaced

**Line 71, remove the word óbviously', suggest to make less definitive statements, add "can cause" and add that this study was in mice**

The sentence now reads:

*LN79: Antibiotics, such as ampicillin, may have a strong impact on GM, which causes decreased abundances of $T_{reg}$ and Th in both mice [31] and humans [32].*

**Line 74, modify that this is a study that was performed in mice**

Done

**Line 76, add that this is for influenza vaccination**

Done

**Line 83, cite literature that shows this**

Done

**Line 88, citation is not about Bangladeshi children**

This has been corrected

*Study Design*

**Line 136, what was the rationale for the different timing of tetanus vaccination between the three studies? Both timing of prime following arrival and timing of boost differed between Study A and B as well as the time of measurement of the immunoglobulin response following the second vaccination. For study A, why were antibodies measured 2,5 weeks after vaccine dose 1 but 3,5 weeks after vaccine dose 2**

This has been clarified and corrected above.

**Line 145 and 151, describe when antibody titers were measured in relation to vaccination**
**Stud C, Line 153, antibody titers are measured 1 week following vaccination, why was this time point chosen? Why was a different interval chosen for duration of diet and antibiotics in relation to stud a and c?**

This has been clarified in a previous comment to the reviewer. We have revised the study plan descriptions accordingly.

**Line 233 – How were titers accounted for in the statistics, why were antibody titers not transformed? Please give rationale or log-transform antibodies/present as geometric mean titers**

Antibody titers were calculated according to the standard curve made up according to the suppliers instructions. This has been put into materials and methods (LN204). The statistics have been recalculated as described above.

**Lines 258/262, list test used for significance**

The tests have already been described in the statistics section.

**Line 259 – please define TM7**

The bacterium used to have the candidate name TM7, but it has been reclassified as Saccharibacteria_ Saccharimonadaceae. This has been put as a footnote in the table.

**Line 263 – Phylum should not be italicized**

Corrected

**Line 269, which microbiome metric is being given a significance here?**

Relative abundances. This has been written in the legend now.

**Line 272, what was the impact of antibiotics on weight in study C**

The mice were not weighed at the end of this study.

**Line 276, missing figure legend for figure 1?**

It has now been inserted (LN127).

**Figure 2, e and f, list time of blood draw in relation to vaccination; list timing of fecal sample collection, list timing of vaccination in figure or figure legend. Correct figure label for e/f to be anti-tetanus IgG**

Done

**Figure 3, list timing of fecal sample collection for microbiome, list timing of IgG in relation to vaccination, list timing of vaccination. Correct figure legends to be anti-tetanus IgG**

**Line 280/285/294 –is this absolute or relative abundance?**

All ‘abundance’ have been replaced with ‘relative abundance’.

**Figure 4e, significance is listed as text but not shown for antibiotics – what has a significance of p= 0.008**

This is a P-value calculated in a two-way ANOVA. The test gives a P-value for each factor, i.e. antibiotics+/- and gluten-free/gluten. The P-value for the factor antibiotics+/- is 0.008 (actually after ranking the data it is now 0.002). There is no significant effect of a gluten-free diet. We cannot really see other ways to write this, and this is also how we normally write the outcome of a two-way ANOVA in papers.

**Table 2, describe duration of time mice were fed diet (4,5 weeks from Study C or 2 weeks from Study A?) Add n for column control and gluten-free**

Done

**Line 309, figure 1 does not show a serum sample 2 weeks after the first vaccination for Study B, please adjust.**

This has been corrected.

**Line 312, what data/statistical test supports the borderline lower variation. Please list statistical test for all p-values.**

This was shown by Levene’s test, which has now been stated.

**Line 316, please indicate which comparison is being made for the significance here**

A two-way ANOVA on ranked data, which has now been stated.

**Lines 321-337, please do not report trends, only significant values**

Point taken. It has been omitted.

**Figure 5, please indicate which comparisons are significant in the figure rather than describing them on the figure.**

As also stated for reviewer No 2, these P-values have been calculated in a two-way ANOVA, in which you get a P-value for each factor. We do not consider it appropriate statistics to do post-hoc tests after a two-way ANOVA, as the increased search for P-values increases the risk of type I error, and the P-values generated by the two-way ANOVA fully responds to the hypothesis. However, if the editor wants us to do it, we will do it and put it into the figures.

**Please indicate how many mice were specifically in each group (e.g. n=10/group, not 10 overall?).**

Done

**Please label the individual plots, rather than 5a/b alone.**

The individual plots are labelled.

**Figure 5B seems to be missing?**

We are not aware in which part of the process this problem has been generated, but it has been submitted with this revision.

**Line 367, suggest to specify IgG with anti-tetanus IgG**

Done

**Line 368, remove reference to 'low-responder' unless this term is defined and tested with a statistical bound.**

Done

**Line 369, suggest to remove dramatic, suggest to specify 'reduced anti-tetanus IgG following the first tetanus vaccination"**

Done

**Line 372, inter-individual variation in GM or antibody response?**

This phrase has been deleted from the restructured discussion.

**Lines 372-374, this sentence's meaning is confusing**

We agree, and it has disappeared in the revised discussion.

**Line 383, these findings were associative, do the authors mean, for future mechanistic explanations?**

Yes, we have now written for future mechanistic studies.

**Line 384/393, suggest correlating bacterial abundance to antibody titer/Treg to support such an association.**

We have correlated all OTU's of the gut microbiota sequencing with the IgG, and it gave a little that we have inserted as a S3 Table. We are not convinced that it makes sense to highlight it very much in the paper.

We write as follows:

*LN273-277: We found a significant correlation after FDR correction of especially species within the phylum Bacteroidetes with the pooled IgG levels of both time points in the antibiotic treated mice compared to their controls (Study B), while no correlations were found after the first vaccination, in singe groups or in study A (S3 Table).*

*LN406-409: We observed a correlation with anti-tetanus IgG in study B, i.e. the antibiotics treated mice. However, this should not be over-interpreted, because in the antibiotics treated group, which had a reduced anti-tetanus*

*IgG production, there was reduced relative abundances of Bacteroidetes, and we found no correlations in study A.*

**Line 395, see earlier comment on definition high/low responder**

We have omitted all talk about high and low responders.

**Please provide a limitations section, in which differences in experimental design between the study arms are taken into account. Also please address the limitation of doing this study in mice and looking only at tetanus vaccine.**

We have inserted a limitations section after the discussion, and removed the last part of the discussion to a conclusions section:

*LN458-471: Studies in mice typically give some basic information, but they are quite often not translatable to humans, i.e. our data cannot be taken as a solid statement that a gluten-free diet has the same impact in humans. Also, there were differences in the experimental setup when testing the impact of a gluten-free diet and the impact of antibiotics. So, although it seemed as if the way these two factors impacted the vaccine responses differed, this interpretation should be made with the precaution that the two studies are not directly comparable. Finally, these studies were performed with a tetanus toxoid vaccine, which globally in humans is known as a highly efficient vaccine [39], and to which the immune system responds in ways, which can be different from responses to other vaccines. The effectiveness of viral vaccines seems to vary far more. Titers to influenza varies significantly between vaccinated humans in different geographical regions [1], and even the most efficient COVID-19 vaccines do not offer full protection against infection in humans [74]. Therefore, our studies in mice should not be used to question the efficiency of tetanus vaccination in humans, and from a more applied point of view it would be equally or more relevant to study the impact of the GM on such vaccines; especially in the light of the COVID-19 pandemic.*

**Please address the potential impact of the metabolome as mediator of a microbiome effect as well as other components of the microbiome, such as virome/fungiome that have not currently been measured.**

We have now inserted the following in the discussion:

*LN439-445: The dietary impact on the microbiome not only relates to the bacterial community of the host. Even specific pathogen free mice harbours a gut virome both in the form of phages [64] and mammal viruses [65]. These may have a major impact on host responses [66]. The microbiomic as well as the host related digestion produces a metabolome, and the*

*components of this may equally well facilitate differences in immune responses [67].*

We do feel that this is a very much improved manuscript, and we thank the reviewers for these valuable inputs. We hope that our manuscript can now be accepted for Plos One.

Yours sincerely,

Axel Kornerup Hansen
Professor, DVM, DVSci
