# Supplementary material for: Effect of gluten-free diet and antibiotics on murine gut microbiota and immune response to tetanus vaccination
Source: PLoS One. 2022 Apr 13;17(4):e0266719. doi: 10.1371/journal.pone.0266719 (PMC9007335; doi:10.1371/journal.pone.0266719)
Supplement: Attachment — Submitted filename: Response to reviewers.pdf [file pone.0266719.s007.pdf]

We have updated the reference:

53. Hansen CHF, Larsen CS, Zachariassen LF, Mentzel CMJ, Laigaard A, Krych L, et al. Gluten-free diet reduces autoimmune diabetes mellitus in mice across multiple generations in a microbiota-independent manner. J Autoimmun. 2022;127:102795. Epub 2022/02/02. doi: 10.1016/j.jaut.2022.102795. PubMed PMID: 35101708.

Reviewer 2 still has some issues with the authors' responses regarding Figure 5 and the goals of the study. I think some of this could be addressed by changing the title to be more reflective of the manuscript content (as is, it is a bit sweeping in nature). I suggest something on the order of: "Effect of gluten-free diet and antibiotics on murine gut microbiota and immune response to tetanus vaccination."

We have changed the title to: ‘Effect of gluten-free diet and antibiotics on murine gut microbiota and immune response to tetanus vaccination’.

I think it would also be good for the authors' to include the Dunnett's post hoc test (in addition to what has already been done) and provide a brief comparative discussion of the results, including caveats to the analyses and limitations of the study design.

We have done Dunnett’s post hoc comparisons on all data in Figure 5 comparing the factorial groups to the ‘Vacc’ group. We have explained this in the statistics section and in the figure legend. We have introduced a sentence under ‘Limitations’: ‘The two-way setup for testing the impact on cell counts does not include a non-vaccinated group, so it is only possible to make conclusions on the relative impact of the experimental factors.’
