# Supplementary material for: Effect of gluten-free diet and antibiotics on murine gut microbiota and immune response to tetanus vaccination
Source: PLoS One. 2022 Apr 13;17(4):e0266719. doi: 10.1371/journal.pone.0266719 (PMC9007335; doi:10.1371/journal.pone.0266719)
Supplement: Attachment — Submitted filename: Response to reviewers.docx [file pone.0266719.s006.docx]

**Reviewer #2:**
*The examination of the immune cell responses in Figure 5 needs to be clarified a little bit more.
1) what is the baseline levels of all the immune cell population without vaccination?
4) Pls indicated which two groups were compared when indicated there is a statistcial significance.*

With due respect to the reviewer, we think that the reviewer has misunderstood the experimental setup. The hypotheses decided upon during the planning of the study were (1) ‘Does gluten have an impact on immune cell populations?’ and (2) ‘Does antibiotics have an impact on immune cell populations’? It is standard within experimental design to answer such two-factorial research questions in a two-factorial setup evaluated by a two-way ANOVA. The set-up looks as follows:

|  |  | Factor 1 Gluten | |
| --- | --- | --- | --- |
|  |  | + | - |
| Factor 2  Antibiotics | + | Group 1 | Group 3 |
|  | - | Group 2 | Group 4 |

The two-way ANOVA will give an individual p-value for each of the factors. Of course, the groups can be compared in a post-hoc test, such as Tukey’s post hoc comparison, but we have no hypothesis relating to differences between the individual groups and the study was not planned for this. The only statistical effect will be that we get more p-values, which we subsequently will have to create hypotheses to explain. This is not good experimental design and statistics.

Alternatively, the study could have been set up for evaluation by a one-way ANOVA, i.e. an unvaccinated group to be compared with different groups with or without gluten and antibiotics. If an overall significant p-value was found in the one-way ANOVA these groups could subsequently be compared with the control group in a Dunnett’s post hoc test. This is, in our eyes, a much weaker design, because it is difficult to interpret the effects of the two factors individually, and it implies multiple testing, which is FDR corrected in the Dunnett’s post hoc test.

However, no matter, which design is preferred, the simple answer is that we planned and did the two-way setup and this cannot subsequently be changed into a one-way setup.

As mentioned in our previous rebuttal letter, if the editor wants us to do the post-hoc test, it can, of course, be done, but in our eyes, it still would not be correct. However, the data in Figure 5 is a minor part of the study, and we do not want them to be a blocker for publication in PLOS One, so we will do as he editor prefers.

*2) why the n number is not consistent (e.g., for Treg n=5, while total CD3 T n=10?)*

For rationalization, we chose to limit the number of mice tested for some of the antibodies. A few cell preparations were unsuccessful, and for those few groups n was reduced accordingly. It was already explained in the previous version of the manuscript in line 226-228: ‘For rationalization some antibodies were only tested on randomly selected mice, and 5, 10 or 15 mice were tested on each level (Figure 5a-b). In some groups some of the preparations were unsuccessful and, therefore, n was reduced accordingly.’

*3) how to explain the cell number change differences between spleen and MLN?*
The mesenteric lymph nodes represent the immune reaction locally in the gut, while the spleen is regarded as an expression of the systemic response. Not all reactions in the gut are translated into a systemic response. This was already explained in line 396 – 399 in the previous version of the manuscript: ‘In the present study we found that the gluten-free diet lowered the levels of T_reg_ locally in the gut, while in the spleen, which should be regarded as an expression of the systemic effect, the gluten-free diet increased T_reg_ fractions, CD4^+^ T cell activation, and tolerogenic dendritic cell fractions and activation, thereby extending the downregulating effect of the T_reg_’_._

*In my opinion, a fecal transfer experiment to demonstrate the effect of gluten-free diet on IgG response is dependent on gut microbiota should be performed to strength this manuscript.*

The reviewer has a point but there is already a large data amount in the present paper, and the paper has been on a long track with the journal. We have already mentioned this in the discussion in the previous version of the manuscript (ln 390-392): ‘Whether the impact on vaccine response is GM dependent might be further elucidated through a fecal matter transplantation to germ-free mice.’ We also refer to another study we have published, while awaiting the progression of the present paper, showing how immune effects of a gluten-free diet can be both microbiota dependent and independent. We are in a process of doing further studies within this subject, and fecal matter transplantation is, of course, relevant. However, we also acknowledge the input from reviewer 3 that we should consider other infectious agents, and, therefore, it is not relevant to put these data into this manuscript.

**Reviewer #3**

*The authors have addressed the majority of my comments and questions*
Thank you.

Small comments:

*(page 54) Abstract: line 32, add lower initial vaccine titer*
Done

*(page 58) line 114: although this vaccine generally has high immunogenicity….*

Done

*(line 161) ‘we reconsidered this’, suggest to include a rationale here*
The sentence now read s’ while we in study B considered that his might be too short and did the boosting after four weeks’.

*(lines 176/ 183) body weights of mice … were monitored. What was the impact of antibiotics and diet on weight in studies A/B (this could be confounding)*
This is the ‘materials and methods’ section, and therefore probably it is not the right place to give results. However, the weight curves are shown in S1 Figure and the interpretation that both diet and antibiotics influence weight are referred to in the first sub-section of ‘Results’ (ln 270-273): ‘Mice fed the Altromin modified gluten-free diet weighed significantly more than mice fed the standard Altromin diet in study A (S1 Figure a, P = 0.000). Antibiotics in the drinking water reduced the weight significantly in study B (S1 Figure b, P = 0.001), but this was mostly due to a weight decrease in the first weeks on antibiotics.’

*(line 314/315) describe the direction of the correlation and clarify if correlation was for the phylum or species level.*
Done

*Line 317 typo, ‘single’*
Done

*Line 347, Table 2 – list all FDRs in table or indicate which FDR level was used to generate table. Relative repeated twice in table legend*
Done

We do feel that our manuscript now should be ready for publication, and we thank the reviewers for their valuable inputs. We hope that our manuscript can now be accepted for Plos One.
